# Finite Element Analysis of Stress Distribution In Tooth-Implant Retained Prostheses: Impact of Periodontal Support, Tooth Count, and Implants: Stress Distribution in Tooth-Implant-Supported Prostheses

**DOI:** 10.31661/gmj.v13iSP1.3608

**Published:** 2024-12-08

**Authors:** Farzan Younesi, Solmaz Soleimanpour, Mohammad Alihemmati, Fatemeh Bakhtiari, Farnaz Taghavi, Maryam Jahangiri, Shojaedin Shayegh

**Affiliations:** ^1^ Prosthodontics department, Dental Faculty, Islamic Azad University of Tehran, Tehran, Iran; ^2^ Department of Prosthodontics, Faculty of Dentistry, Shahed University, Tehran, Iran; ^3^ Department of Prosthodontics, Faculty of Dentistry, Shahid Beheshti University of Medical Sciences, Tehran, Iran

**Keywords:** Tooth-Implant-Supported Prosthesis, Finite Element Analysis, Periodontal Support, Stress, Von Mises Stres

## Abstract

**Background:**

The durability of tooth-implant supported restorations prostheses is significantly influenced by biomechanical factors. Despite advancements in dental implant technology, integrating natural teeth with implants remains a challenge due to their disparate biomechanical properties. This study employed 3D finite element analysis to investigate the impact of periodontal support and the number of teeth and implants on stress distribution within these prostheses.

**Materials and Methods:**

Six virtual models of tooth-implant retained prostheses were constructed using 3D finite element analysis. These models varied in terms of periodontal support (normal and compromised) and bridge design (three-unit, four-unit with two dental abutments, and four-unit with two implants). A patient’s CBCT scan provided the basis for a realistic mandibular bone model. A single ITI implant was utilized, and the teeth and bridge frameworks were designed according to standard metal-ceramic prosthesis principles. Static forces of 250 N were applied vertically and obliquely to assess stress distribution, measured in megapascals (MPa).

**Results:**

Compromised periodontal support significantly increased stress on the implant and bone, particularly under oblique loading. Specifically, stress levels increased by approximately 21% under vertical loading and 24-25% under oblique loading. Conversely, increasing the number of teeth and implants substantially reduced stress on the implant and bone. Oblique forces consistently induced higher stress levels compared to vertical forces.

**Conclusion:**

This study indicates that teeth with a 1:1 crown-to-root ratio are optimal abutments. To minimize stress and reduce the risk of complications, increasing the number of teeth and implants, along with appropriate occlusal adjustments, is recommended.

## Introduction

Advancements in implantology have significantly improved treatment options for partial edentulism [1]. One of the challenges in treating partial edentulism is effectively integrating natural teeth with implants in tooth-implant retained prostheses. Previous studies have highlighted that misalignment between natural teeth and implants can lead to increased stress on the implant and the entire prosthetic system, potentially causing complications such as tooth intrusion, bone loss, screw loosening, and prosthesis or implant failure [2]. To mitigate these risks, fully implant-supported prostheses are often preferred [3][4]. However, in specific clinical scenarios, such as in Division C-h ridge scenarios in the pontic region, or when using narrower diameter or distally positioned posterior implants, tooth-implant retained prostheses can be considered a viable alternative. Despite the potential benefits and comparable success rates to fully implant-supported prostheses [5][6], optimizing the design and clinical application of tooth-implant retained prostheses requires a thorough understanding of their biomechanical behavior.

It has been noted that using non-rigid connectors in tooth-implant retained prostheses can improve stress distribution in the peri-implant bone. However, this may not enhance stress distribution within the prostheses themselves, potentially increasing the risk of dental intrusion [7][8]. Consequently, most studies recommend rigid connectors [2][3][6][9][10]. Factors such as connector type and location, force magnitude and direction, implant design and number, and periodontal support significantly influence stress distribution and the risk of biomechanical complications in tooth-implant retained prostheses [11]. Compromised periodontal support can exacerbate tooth movement and bending loads, further complicating the biomechanical behavior of tooth-implant retained prostheses [9]. Studies have reported a correlation between bone height and stress distribution [3][9].

Given the major role of biomechanical factors in the durability of tooth-implant supported restorations prostheses [12], and the limited information provided by clinical experimental studies, various methods, such as photoelastic techniques, finite element analysis, and strain gauge analysis, are currently employed to evaluate stress distribution and biomechanical behavior in these prostheses [13]. Finite element analysis, in particular, offers precise information about force distribution through the creation of virtual models. This method has become increasingly valuable as a supplementary tool for assessing biomechanical responses in dental studies [14]. Considering the crucial role of biomechanical factors and the conflicting findings in existing research regarding the impact of periodontal support and the number of abutments, this study aimed to evaluate how periodontal support, the number of teeth, and splinted implants affect stress distribution in tooth/implant-supported prostheses using three-dimensional (3D) finite element analysis

Implantology advancement offer better treatment choices for partial edentulism. tooth-implant retained prostheses offer a viable solution, but their long-term success is influenced by various factors, including periodontal support, the number of teeth and implants, and the direction of occlusal forces. This study aims to address the following research questions:

How does periodontal health affect stress distribution in tooth-implant retained prostheses?

How does the number of teeth and implants influence distribution of stress force?

How does the direction of biting forces (vertical vs. oblique) affect distribution of stress force?

By answering these questions, we hope to provide evidence-based recommendations for designing and clinically applying tooth-implant retained prostheses effectively. We hypothesize that increasing the number of teeth and implants, while maintaining optimal periodontal health, will significantly reduce stress on the implant-bone interface, leading to improved long-term clinical outcomes.

## Materials and Methods

**Figure-1:**
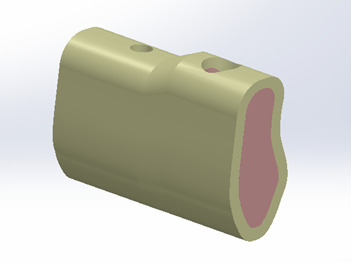
Figure 1. Creating the required height difference along the mesiodistal distance to model the tooth-implant-supported bridges

This study utilized 3D finite element analysis to evaluate six models. Three mandibular metal-ceramic posterior bridges were designed with two types of periodontal support, including normal (1 mm below the cementoenamel junction (CEJ)) and compromised (crown-to-root ratio of 1:1). Table-1 and Figure-1 details the specifications of these models.

## Modeling Process

**Table:** Table1. The studied three-dimensional models.

Model number	Acronym*	Tooth-implant-supported prosthesis design	The abutments	Periodontal support
1	3N	3-unit bridge	First premolar (tooth) Second premolar (pontic) First molar (implant)	Normal
2	4(2t)N	4-unit bridge	First premolar (tooth) Second premolar (tooth) First molar (pontic) Second molar (implant)	Normal
3	4(2i)N	4-unit bridge	First premolar (tooth) Second premolar (pontic) First molar (implant) Second molar (implant)	Normal
4	3C	3-unit bridge	First premolar (tooth) Second premolar (pontic) First molar (implant)	Compromised
5	4(2t)C	4-unit bridge	First premolar (tooth) Second premolar (tooth) First molar (pontic) Second molar (implant)	Compromised
6	4(2i)C	4-unit: First premolar - two implants in distal- weak periodontal support	First premolar (tooth) Second premolar (pontic) First molar (implant) Second molar (implant)	Compromised

^*^ **N:** Normal, **C:** Compromised, **t:** Tooth, **i:** Implant

### Model Design

Six virtual models of tooth-implant retained prostheses were created using SolidWorks 2014. The models varied in terms of periodontal support (normal and weak) and bridge design (three-unit, four-unit with two dental abutments, and four-unit with two implants).

### Tooth and Implant Models

Tooth dimensions were based on standardized dental anatomy (Wheeler’s dental anatomy). ITI implants (Straumann, Switzerland) (4.1 × 10 mm) with a straight abutment (Art No. 048.605) were used as the implant model.

### Periodontal Ligament

A 0.3 mm periodontal ligament was simulated the soft tissue connection between the tooth and bone [16].

### Prostheses Properties

A 3 × 3 mm rigid connector, 0.5 mm minimum metal framework thickness, and 1-2 mm feldspathic porcelain thickness were used. A noble metal alloy was chosen for the framework, with a premolar serving as the pontic.

### Patient Selection and CBCT Data:

Cone Beam Computed Tomography (CBCT) scans were acquired from a middle-aged patient with good oral health and suitable bone density. A detailed virtual 3D mandibular bone was generated from CBCT data. To simulate normal and compromised periodontal support, the bone model was modified by adjusting the position of the teeth and implants relative to the cementoenamel junction (CEJ). Six models were generated to represent different combinations of periodontal support and bridge design [17].

A modified bone model was created to simulate normal and poor periodontal support for bridges. The bone was adjusted to position it either 1 mm or 3 mm below the CEJ, depending on the support. Tooth roots and fixtures were placed accordingly, ensuring proper placement and support. Six models were completed in total.

## Meshing Process

**Figure-2:**
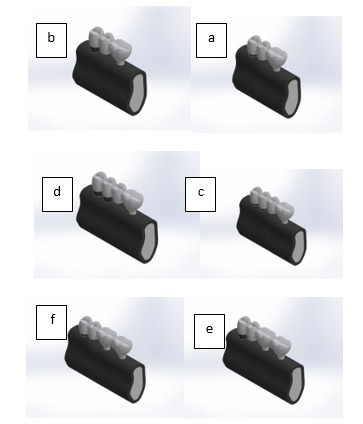
Figure 2. A 3-unit bridge-base tooth: First premolar – one implant in distal- weak periodontal support; B: A 3-unit bridge-base tooth: First premolar – one implant in distal- normal periodontal support; C: A 4-unit bridge-base tooth: First and second premolar – one implant in distal- weak bone support; D: A 4-unit bridge-base tooth: First and second premolar – one implant in distal- normal periodontal support; E: A 4-unit bridge-base tooth: First premolar – two implants in distal- normal periodontal support; F: A 4-unit bridge-base tooth: First premolar – two implants in distal- weak periodontal support.

**Table:** Table2. The number of nodes and elements in the six models.

Model number	Acronym	Number of nodes	Number of elements
1	3N	25272	132350
2	4(2t)N	30559	161222
3	4(2i)N	34536	180032
4	3C	24375	127168
5	4(2t)C	29624	155673
6	4(2i)C	33903	176414

Meshing was performed on all models using hexagonal solid elements. Table-2 and Figure-2 show the number of elements and nodes for each model. The fixture-to-bone attachment was modelled as bonded in this study, representing 100% osteointegration [3][9][16][18]. Additionally, the fixture-to-abutment connection was considered to be bonded, preventing slipping between them [18].

### Material Properties and Loading

The physical properties of the materials, such as modulus of elasticity and Poisson ratio, were input into the software based on values reported in relevant literature (Table-3). All materials were assumed to be isotropic, homogeneous, and linear elastic [3][9][16][19][20].

The models in this study were loaded in two modes: 1) The vertical static force of 250 N [21] was applied to a small circular surface with a diameter of 0.5 mm at the center of the central groove of the pontic and tooth retainer (premolar crown), and at two similar locations on the implant retainers (molar crown), each 3 mm from the marginal ridge. 2) The static force of 250 N at a 45 degree angle to the longitudinal axis [22] was applied to a circular surface with a diameter of 0.5 mm on the buccal incline of the buccal cusp of the pontic and dental retainer, slightly lower than the cusp tip, and on two similar locations on the implant retainers. The force direction was from buccal to lingual. The applied force of 250 N was considered a balanced force, not the maximum bite force.

### Model Validation

To ensure the accuracy and reliability of the virtual models, the following steps were taken:

Geometric Accuracy: The 3D models were meticulously created using precise measurements and anatomical landmarks. The accuracy was verified by comparing the models to clinical images, such as CBCT scans.

Material Property Validation: The material properties assigned to the different components (bone, teeth, implants, and connectors) were based on well-established values from the literature. Sensitivity analysis was performed to assess the impact of variations in material properties on distribution of stress force.

Boundary Conditions and Loading: The boundary conditions and loading conditions were applied to simulate real-world clinical scenarios. The magnitude and direction of the applied forces were based on previous studies and clinical observations [21][22].

Comparison to Existing Research: While direct experimental validation is challenging, the finite element analysis results were compared to findings from previous studies and clinical observations [17].

By incorporating these validation steps, we aim to ensure the accuracy and reliability of the virtual models in predicting the biomechanical behavior of tooth-implant retained prostheses.

### Stress Analysis

To evaluate the mechanical integrity of the models under the applied loads, the maximum Von Mises stress was calculated for each element using Cosmos 2014 software. Von Mises stress is a comprehensive measure that accounts for both normal and shear stresses.To compare distribution of stress force among different models, statistical analysis was performed. One-way ANOVA was used to compare models with varying periodontal support and bridge designs under specific loading conditions. Two-way ANOVA was used to assess the combined effect of periodontal support and bridge design on distribution of stress force. Tukey’s HSD test was used to identify significant pairwise differences between groups. Statistical significance was determined at a significance level of α = 0.05 (Table-4and5).

## Results

**Figure-3:**
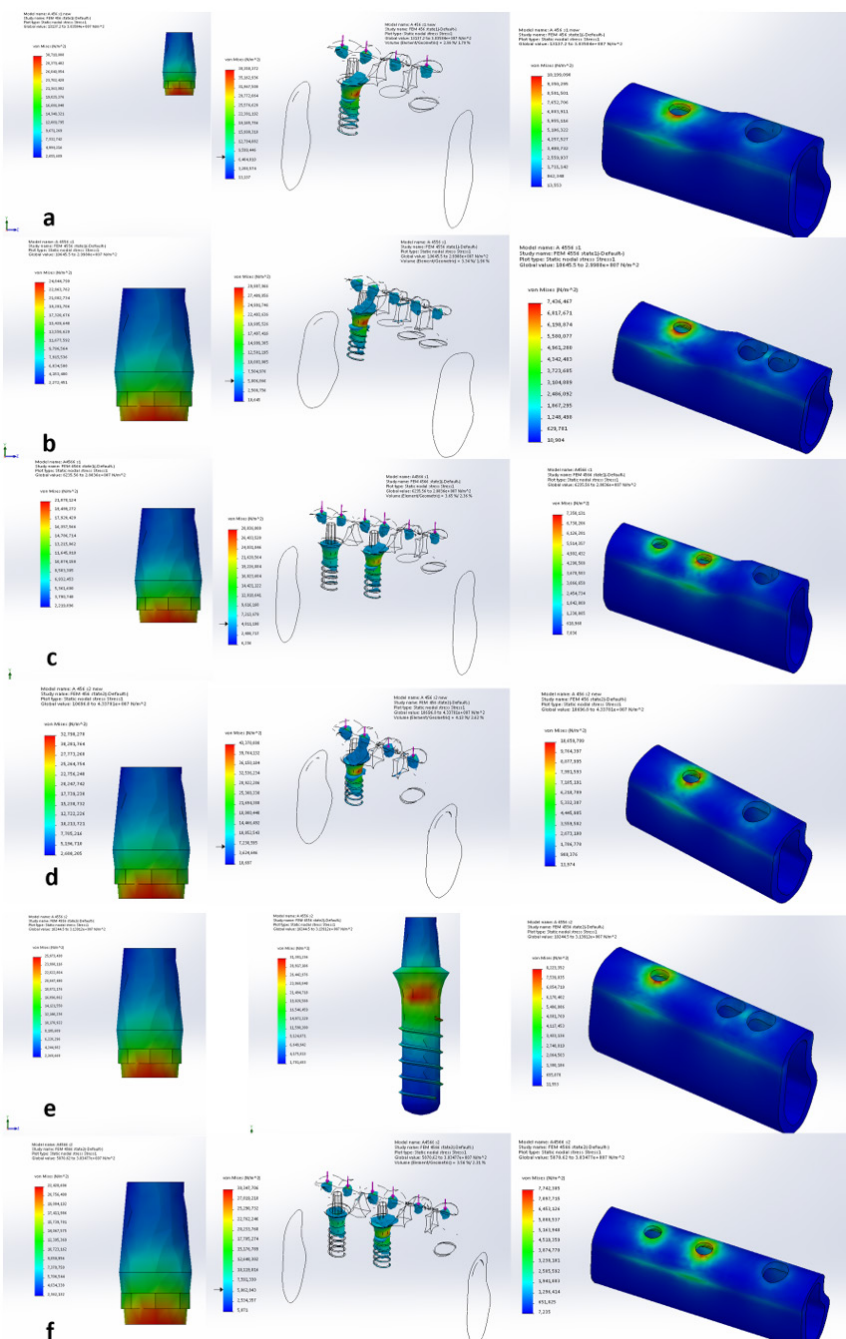
Figure 3. Von Mises Stress distribution at structure components when applying vertical force.

**Figure-4:**
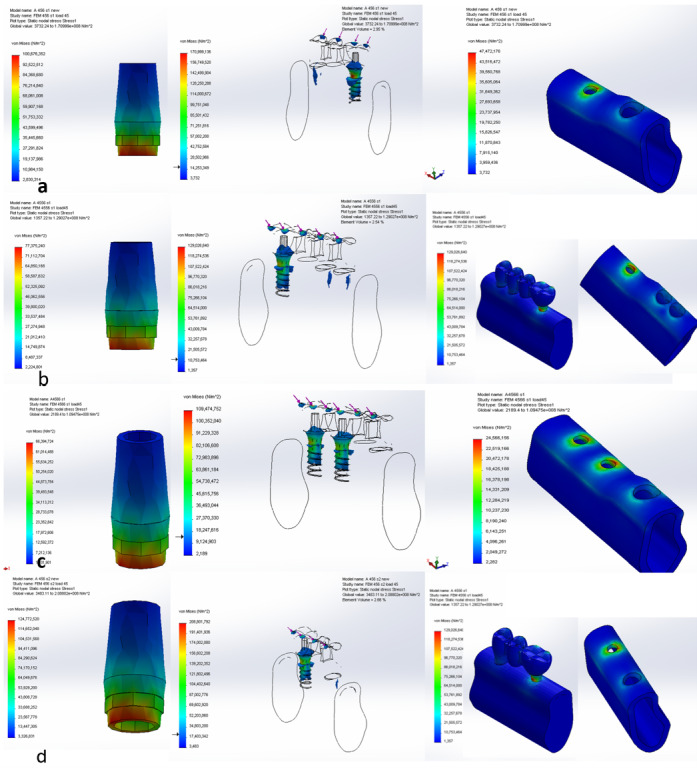
Figure 4. Von Mises Stress distribution at structure components when applying oblique force.

**Table:** Table3. The physical properties of the materials.

Material name	Elastic Modulus (MPa)	Poisson ratio
Titanium (fixture-abutment)	110	0/35
Gold (framework)	100	0/3
Porcelain	69	0/28
Dentin	18/6	0/31
Periodontal ligament	69	0/45
Cortical bone	15	0/3
Trabecular bone	1/5	0/3

**Table:** Table4. Maximum Von Mises Stress under the vertical forces (MPa).

Model number	Acronym	Fixture	Distal fixture	Abutment	Cortical bone	Trabecular bone
1	3N	38/358	-	30/718	10/199	4/95
2	4(2t)N	29/988	-	24/845	7/436	4/521
3	4(2i)N	28/836	17/583	21/07	7/35	3/557
4	3C	43/378	-	32/799	10/651	5/972
5	4(2t)C	31/391	-	25/973	8/223	4/239
6	4(2i)C	30/348	18/980	22/429	7/742	4/651

**Table:** Table5. Maximum Von Mises Stress under the oblique force (MPa).

Model number	Acronym	Fixture	Distal fixture	Abutment	Cortical bone	Trabecular bone
1	3N	170/999	-	100/676	47/472	14/366
2	4(2t)N	129/029	-	77/375	30/448	12/026
3	4(2i)N	109/476	101/822	66/395	24/566	9/927
4	3C	208/802	-	124/773	50/672	20/545
5	4(2t)C	163/258	-	99/501	32/953	13/026
6	4(2i)C	128/175	111/338	80/134	27/987	11/456

The distribution of stress force pattern under vertical force was consistent across all six models. Maximum stress concentration occurred at the interface between the coronal part of the fixture and the cortical bone, notably affecting the buccal and lingual parts of the bone. Stress levels in the trabecular bone were notably lower compared to the cortical bone. In models with two implants, the stress magnitude and extension to the apical part of the fixture were reduced in the distal fixture (farther from the pontic) (Figure-3).

The distribution of stress force pattern under oblique force (45°) is largely similar to that under vertical force, but the location of maximum stress concentration shifts to the lingual side instead of the mesial side. In models with two implants, there is minimal difference in distribution of stress force and its extension towards the apical part between the distal fixture and the primary fixture. Stress levels, especially in the fixture and cortical bone, are higher under oblique force compared to vertical force. Increasing the number of teeth and implants results in a notable reduction in stress across all components, especially in the fixture and abutment, with slightly greater benefits observed in models with compromised periodontal support(Figure-4).

On the other hand, applying the oblique force showed that poor periodontal support both 3-unit or 4-unit bridge designs increased stress across all components. Increasing the number of abutments significantly reduced stress in areas such as the fixture, abutment, cortical bone, and trabecular bone. Moreover, incorporating implants resulted in greater stress reduction compared to using tooth abutments.

The results of the finite element analysis revealed significant differences in distribution of stress force across the various models.

### Periodontal Support

Models with compromised periodontal support exhibited a 21% increase in maximum Von Mises stress on the implant and surrounding bone compared to models with normal periodontal support. This difference was statistically significant (P < 0.05) (Table-6).

### Number of Teeth and Implants

Increasing the number of teeth and implants significantly reduced stress on the implant and bone. The four-unit bridge with two implants showed a 25% reduction in maximum stress compared to the three-unit bridge. This difference was statistically significant (P < 0.01) (Table-6).

### Direction of Force

Oblique forces induced significantly higher stress levels than vertical forces. The maximum Von Mises stress was 400% higher under oblique loading compared to vertical loading (P < 0.001) (Table-6).

The results of the finite element analysis revealed significant differences in distribution of stress force across the various models (Table-6).

## Discussion

**Table:** Table6. Summary of Maximum Von Mises Stress (MPa) for Different Models

Model	Vertical Load (MPa)	Oblique Load (MPa)
3-Unit Bridge (Normal)	38/358	170/999
3-Unit Bridge (Weak)	29/988	129/029
4-Unit Bridge (2 Teeth, Normal)	28/836	109/476
4-Unit Bridge (2 Teeth, Weak)	43/378	208/802
4-Unit Bridge(2Implants, Normal)	31/391	163/258
4-Unit Bridge(2 Implants, Weak)	30/348	128/175

Our findings showed that vertical force consistently resulted in maximum stress at the interface between the coronal part of the fixture and the cortical bone, particularly on the mesial side, with lower stress levels in the trabecular bone. Models with two implants showed reduced stress magnitude and extension to the apical part in the distal fixture. Under oblique force, the stress concentration shifted to the lingual side, with higher stress levels compared to vertical force, especially in the fixture and cortical bone. Poor periodontal support in three-unit bridges remarkably increased stress levels, while four-unit bridges showed only a slight increase. Increasing the number of teeth and implants notably reduced stress, with greater benefits in compromised periodontal support models. Implant abutments provided slight advantages over tooth abutments in stress reduction.

Tooth-implant-supported prostheses are recommended when no other fixed restoration options are available. They provide a good immediate and sustained solution with fewer incidences of failure. Narde et al., in their clinical study, concluded that tooth-implant retained prostheses show a very good survival rate [23]. Our study used 3D finite element analysis to investigate the stress in the implant and surrounding bone in three designs of tooth-implant retained prostheses: a 3-unit bridge, a 4-unit bridge with 2 tooth abutments, and a 4-unit bridge with two implants. We examined two conditions of periodontal support (normal, crown-to-root ratio 1:1) under vertical and oblique loads. According to the Von Mises Stress results, the pattern of bone distribution of stress force did not significantly differ between models with normal or weak periodontal support and different bridge designs. The only notable difference was in the stress values.

In this study, the models were designed accurately in accordance with accepted standards. The CBCT of a patient with normal bone contour was used to build a bone model, providing a more realistic representation compared to the cubic bone block design used in some studies. Despite some simplifications, this method produced more accurate results [3][9].

In this study, a prosthesis with a rigid connector was considered. As a result, the stress around the natural teeth and implants was reduced, ensuring long term stability [24]. On the other hand, Nitin et al. found that using non-rigid connectors in tooth-implant retained prostheses is advisable, particularly placing the non-rigid connector between the implant and the pontic [25]. Similarly, Huang et al. recommended using flexible non-rigid connectors and reported that bridge span distance affects maximum stress[14]. In another study, it was suggested that using a rigid connection for a bridge span of 12 mm, while a non-rigid connection could be used for spans longer than 18 mm [26]. Contrarily, Mosharraf et al. reported that the amount of intrusion is not dependent on the type of connector used, whether rigid or non-rigid [27].

In this study, a prosthesis with a implant-tooth abutment was considered to support fixed partial dental crowns. Verma et al., in a study on the comparison of distribution of stress force with different combinations of support, reported that stress concentration around the implant-bone interface was the highest. The least stress was observed in the bone around the natural tooth due to the dampening effect of the periodontal ligament. The authors suggest that the clinically appropriate combination of abutments should be considered for a fixed partial prosthesis [28].

Oblique force is closer to the reality of functional force and causes stress concentration in the cortical bone around the implant. Some studies, including the study by Narde et al., concluded that oblique forces create greater stress [29]. Accordingly, in this study, applying a static force of 250 N vertically simulated the contacts at maximum intercuspation, while the oblique force simulated the lateral working side contacts. The forces were applied to all parts of the prosthesis (i.e., retainers and pontics).

The maximum bone stress was observed at the interface of the fixture neck, consistent with previous research [16][21][30]. Vertical forces applied to the prosthesis induce tooth intrusion and generate a bending moment in the mesial aspect of the implant. This bending moment causes the implant to rotate, with its center of rotation typically located crestally, higher than the center of rotation for the tooth. This disparity leads to stress accumulation in the crestal bone [31]. In addition, the difference in structure and hardness between cortical and trabecular bones, coupled with their varying modulus of elasticity, makes the cortical bone more susceptible to stress concentration [31].

In this study, distribution of stress force analysis included evaluation of the abutment and prosthesis, in addition to the bone and fixture. Maximum Von Mises stress in the abutment was concentrated at the point of fixture connection and its mesial aspect. In the prosthesis, stress was concentrated at the fixture-abutment connection and extended somewhat distally to the pontic connector. Among the studied variables, such as force direction, periodontal support, and the number of tooth and implant abutments, the direction of force application had the greatest impact on stress generation, consistent with previous research findings [3][16][18][21]. In contrast, within the range tested in this study, reduced periodontal support (crown-to-root ratio 1:1) minimally affected stress levels in the implant and surrounding bone under vertical force conditions. However, reduced periodontal support increased stress levels across all bridge designs, particularly under oblique force conditions.

The present study demonstrated that adding dental abutment significantly reduced stress in the implant and surrounding bone under bone vertical and oblique force applications, with slightly greater stress reduction observed in models with reduced periodontal support. This finding aligns with previous research, such as the study conducted by Dalkiz and colleagues, which showed that increasing the number of splinted teeth decreases bone stress [32]. Similarly, de Oliveira et al. found that more dental abutments lead to reduced stress on prosthesis components [33]. Moreover, multiple studies have highlighted that increasing the number of dental abutments reduced the risk of intrusion [4][8][34]. Another study determined the importance of using at least two teeth as abutments to prevent intrusion in tooth-implant retained prostheses [4]. However, the findings of some other studies are somewhat contradictory to these results [3][9][16]. In this regard, the results of one study showed that adding a tooth to a tooth/implant-supported prosthesis does not provide significant advantages [18], possibly due to differences in methodology, such as using two-dimensional finite element analysis and lower applied forces (10, 20, and 50 N) compared to our study.

Lin et al. reported that adding a dental abutment decreases stress in the implant and bone, particularly in models with reduced periodontal support [9]. However, the present study observed a significant reduction in stress even in models with normal periodontal support when adding a dental abutment. The abovementioned study differed in methodology, used two-dimensional analysis, and focused only on vertical forces with lower applied forces (50 N), which may explain some discrepancies with the present findings. Another study suggested that adding a dental abutment reduced bone stress only under oblique forces [3]. However, the current study found that adding a dental abutment reduced stress in both vertical and oblique force applications. Furthermore, another study by Lin et al. suggested that an additional dental abutment did not significantly alter stress in the implant and bone [16]. These variations underscore the importance of considering different study methodologies and force applications when interpreting results across studies.

The present study has several limitations that should be considered. Firstly, the assumption of homogeneous, elastic, and isotropic properties for bone, teeth, and periodontal ligaments may not fully replicate clinical variability. Moreover, the assumption of 100% osteointegration of implants does not reflect real-world conditions where varying degrees of integration may occur. Secondly, the study applied vertical and oblique static forces as simplified representation of the complex function forces in the oral cavity, potentially limiting the generalizability of the findings. Thirdly, the study which focused on distribution of stress force may overlook other important factors influencing prosthesis longevity and patient outcomes, such as material wear, clinical maintenance, and patient-specific oral hygiene practices. Therefore, while this study provides insights into the biomechanical aspects of tooth/implant-supported prostheses under average conditions, further long-term clinical studies are necessary to establish more definitive conclusions.

## Conclusion

This study investigates the biomechanical factors affecting the success of tooth-implant retained prostheses. Our findings highlight the critical role of periodontal support, the tooth and implants count, and the direction of occlusal forces in determining distribution of stress force and potential complications.

1- Compromised periodontal support significantly increases stress on the implant-bone interface, particularly under oblique loading.

2- Increasing the number of teeth and implants effectively reduces stress on the implant and bone.

3- Oblique forces induce significantly higher stress levels compared to vertical forces.

These findings have significant implications for clinical practice. Clinicians should prioritize maintaining optimal periodontal health, consider increasing the number of abutments, and optimize occlusal schemes to minimize stress and improve the durability of tooth-implant supported restorations prostheses.

Future research should focus on investigating the impact of patient-specific factors, such as bone quality and quantity, on distribution of stress force. Additionally, long-term clinical studies are needed to validate the findings of this study and assess the long-term outcomes of different treatment approaches. By advancing our understanding of the biomechanical principles underlying tooth-implant retained prostheses, we can develop more effective and durable treatment strategies.

## Conflicts of Interests

The authors declare that they have no conflict of interests.
